# Most of the extant mtDNA boundaries in South and Southwest Asia were likely shaped during the initial settlement of Eurasia by anatomically modern humans

**DOI:** 10.1186/1471-2156-6-41

**Published:** 2005-08-04

**Authors:** Mait Metspalu, Toomas Kivisild, Ene Metspalu, Jüri Parik, Georgi Hudjashov, Katrin Kaldma, Piia Serk, Monika Karmin, Doron M Behar, M Thomas P Gilbert, Phillip Endicott, Sarabjit Mastana, Surinder S Papiha, Karl Skorecki, Antonio Torroni, Richard Villems

**Affiliations:** 1 Institute of Molecular and Cell Biology, Tartu University, Tartu, Estonia; 2 Bruce Rappaport Faculty of Medicine and Research Institute, Technion and Rambam Medical Center, Haifa, Israel; 3 Dipartimento di Genetica e Microbiologia, Università di Pavia, Pavia, Italy; 4 Department of Human Sciences, Loughborough University, Loughborough, UK; 5 Department of Human Genetics, University of Newcastle-upon-Tyne, UK; 6 Ecology and Evolutionary Biology, The University of Arizona, Tucson, Arizona, USA; 7 Henry Wellcome Ancient Biomolecules Centre, Department of Zoology, University of Oxford, Oxford OX1 3PS, UK

## Text

After the publication of the paper [1] we have noticed that we have mistyped haplogroup M2 defining mutation in three places in the paper (listed below). It should be 447G instead of 477G.

1. Background: paragraph 2, line 8.

2. The package of the most ancient mtDNA haplogroups in India: paragraph 1, line 3.

3. Table 3: Row 1

We regret any inconvenience that this inaccuracy might have caused. We wish to thank Dr. Gyaneshwer Chaubey for bringing this matter to our attention.
